# The Southern Surgical and Gynecological Association

**Published:** 1891-12

**Authors:** 


BUFFALO MEDICAL AND SURGICAL JOURNAL
A MONTHLY REVIEW OF MEDICINE AND SURGERY.

*EDITORS:*
THOMAS LOTHROP, M. D. - - WM. WARREN POTTER, M. D.

All communications, whether of a literary or business character, should be addressed to the managing editor: 284 FRANKLIN STREET, BUFFALO, N. Y.

VOL. XXXI. DECEMBER, 1891. No. 5.

### THE SOUTHERN SURGICAL AND GYNECOLOGICAL ASSOCIATION.

THE fourth annual meeting of this renowned association was held in Richmond, Va., November 10, 11, and 12, 1891, and the deeds that it there accomplished have already passed into history. There are some features of medical associations that are proper subjects for discussion in the medical journals, and it is one of the provinces of the journalist to advise his readers as to the value of the several medical organizations and the quality of the work they do, while the meetings are still fresh in his mind. It is a pleasure to speak of such a gathering as convened at Richmond last month, because it was composed of some of the most eminent surgeons and gynecologists in the country, and, because further, that the scientific work accomplished was second to that of no special medical organization in the United States.

In the first place, everything conspired to make this meeting one of unusual success; its president was a man gifted in his profession and experienced in affairs; its secretary was indefatigable, energetic, and capable; the chairman of the committee of arrangements was one of the most eminent men that the South has ever produced; the time and place of meeting were the happiest possible — the capital of the Old Dominion, and the season was the loveliest of all the year, the charming Indian summer, that makes Richmond and its environs one of the most delightful spots on the continent. Finally, the weather, that has been charged with failure so many times, was a great and pronounced success on the occasion referred to. The sun shone out in all its softness, the air was mellow with the ripeness of autumn, the foliage with all its changing hues, and the smokiness of Indian summer weather, all these conspired to make the meeting what it should be,—one of great intellectual effort, and of conspicuous results in advancing the interests of the profession.

It would not be just to our Richmond confrères if we did not tell the world something of the way they received the members and guests of this association. It was literally with open arms that they welcomed them, not only to their charming city, but to their homes and firesides ; for the members, in one way or another, were entertained individually or collectively in the drawing-rooms and at the dinner tables of a large number of the resident members of the medical profession. We do not wish to individualize or to speak invidiously, but if we should fail to mention the hospitalities of Dr. Hunter McGuire, Dr. George Ross, and Dr. Geo. Ben. Johnston, we should fail to do justice to our own feelings at least ; while the banquet tendered by the entire profession of Richmond, on Thursday night, was a fitting *finale* to a most remarkable week of work and pleasure. Some of the speeches made at the banquet deserve to be preserved in permanent form, because they were unusually witty, bright, entertaining, and instructive.

We must not forget to mention the many kind and delicate attentions to the visitors by Messrs. Carrington and Archer, of the Exchange and Ballard hotels. It is so unusual for guests to receive such personal care as these gentlemen gave to their patrons, that we feel it deserving of record. The Westmoreland Club, too, kept its doors wide open to the visitors.

We regret that we have not space to go more into the details of this meeting, but we close with the assertion that it was one of the best ever held in America. No one who attended it, or who is in the habit of attending other meetings, will dare gainsay this.

The Association honored itself in electing as its president, for the ensuing year, Dr. J. McFadden Gaston, of Atlanta, Ga., and as vice-president, Dr. Cornelius Kolloch, while the Association concluded to take the risk of electing the secretary and treasurer, Dr. W. E. B. Davis and Dr. H. P. Cochrane, of Birmingham, for another year.

The next meeting was appointed to be held in Louisville, Ky., on the second Tuesday of November, 1892.

---

A NEW REMEDY FOR WHOOPING-COUGH is ouabaine, in doses of about gr. 1-1000. It has been tried with success by Dr. Gemmell, of Glasgow, and, lately, by Dr. J. L. Porteous, of Yonkers, N. Y.—*New York Medical Journal.*

